# Efficacy of music therapy for depressive symptoms in college students: a meta-analysis and systematic review

**DOI:** 10.3389/fpsyg.2025.1576381

**Published:** 2025-07-18

**Authors:** Yanru Lin, Qianlong Li

**Affiliations:** ^1^Department of Music, The School of Music and Dance, College of Chinese & ASEAN Arts, Chengdu University, Chengdu, China; ^2^Department of Pharmaceutical Sciences, The School of Pharmacy, Wuhan University, Wuhan, China

**Keywords:** music therapy, depression, college students, mental health, psychosocial intervention

## Abstract

**Introduction:**

In China, Depression is a significant mental health issue among college students, with a 28.4% prevalence rate. This meta-analysis systematically synthesizes empirical data to evaluate the therapeutic efficacy of music therapy in mitigating depressive symptoms within collegiate populations.

**Methods:**

Two authors searched seven international and Chinese databases from inception to February 5, 2025, for randomized controlled trials (RCTs) on music therapy for college students with depressive symptoms. Statistical synthesis was performed with RevMan 5.3 software, using random-effects models to calculate standardized mean differences (SMDs) with 95% confidence intervals.

**Results:**

Sixteen RCTs involving 938 participants were included. Pooled estimates showed significant reductions in depressive symptoms as measured by SDS (SMD = −1.19, 95% CI: −1.56 to −0.82; *p* < 0.001), SCL-90 depression subscale (SMD = −1.29, 95% CI: −1.96 to −0.62; *p* = 0.002), and BDI (SMD = −1.98, 95% CI: −3.59 to −0.38; *p* = 0.015). High heterogeneity (*I*^2^ = 76–89%) was found, but the results remained robust even after undergoing sensitivity analyses.

**Conclusion:**

Music therapy is an effective adjunctive intervention for managing depressive symptoms in college students. However, due to significant heterogeneity, future research needs standardized treatment frameworks. Multi-center RCTs with blinded assessment and longitudinal designs should be prioritized to clarify dose-response relationships and neurophysiological mechanisms, facilitating the integration of music therapy into evidence-based mental health strategies for this population.

## 1 Introduction

The Institute of Psychology under the Chinese Academy of Sciences carried out a survey on the mental health conditions of nearly 80,000 college students across 31 provinces in China. The survey revealed that the detection rates of depression risks were ~21.48% (Report on the Mental Health of Chinese Nationals, 2022). Depression is a complex mental disorder. According to the World Health Organization (WHO) report, Depression is currently the second leading cause of global disability and is projected to become the primary cause by 2030 (Malhi and Mann, 2018). College students are at an important turning point in their lives. They face academic competition, social confusion, and confusion in career planning. The incidence of depression among Chinese college students is 28.4%, while it is only 5–6% in the general population (Song et al., 2020). In the past few years, the incidence of depressive symptoms among college students has been on the rise (Yan and Zhang, 2024), which undoubtedly brings a heavy burden to both individuals and society. At the individual level, their learning efficiency is significantly reduced, their social circles are constantly shrinking, and they seriously lack self–identity (Yuan et al., 2024). In severe cases, it may even lead to tragic suicides. From a social perspective, this not only means an interruption in talent cultivation but also brings great pain to families, thus affecting social harmony and stability (Ding et al., 2024). Therefore, improving and treating depressive symptoms is of great significance to the college student group and society.

Currently, there are many drugs for treating depressive symptoms in clinical practice. Although these drugs can control depressive emotions to a certain extent, long-term use can easily lead to drug resistance, and recurrence is likely after stopping the medication (Hollon et al., 2002; Liu et al., 2022). Non-drug treatments can effectively stimulate positive emotions without bringing other health risks. Music therapy, as one of the non-drug treatment methods, can stimulate the brain to secrete neurotransmitters, effectively regulate emotions, and relieve depressive symptoms (Legge, 2015; Arnold, 2014; Carr et al., 2023). At present, many researchers have evaluated the psychological and physiological effects of music therapy on patients with depression (Parker and Student, 2019; Xinyi et al., 2023; Pasiori et al., 2024; Lu, 2024). However, the sample size of college students in various studies is relatively small, and the research results vary. Although there have been some Meta-analyses on the impact of music therapy on the depressive emotions of college students (Yu et al., 2020; Ji et al., 2015; Yan et al., 2020, 2019), with the changes in the educational environment and the mental health problems of college students, it is still necessary to update relevant Meta-analysis studies. Therefore, this study uses Meta-analysis to comprehensively evaluate the intervention and improvement effects of music therapy on the depressive emotions of college students, providing an evidence-based basis for the mental health of college students.

## 2 Methods

The present systematic review and meta-analysis were conducted in accordance with the Preferred Reporting Items for Systematic Reviews and Meta-Analyses (PRISMA) 2020 guidelines (Sarkis-Onofre et al., 2021). The study protocol was prospectively registered in PROSPERO (ID: CRD420251072407) to ensure methodological transparency.

### 2.1 Literature retrieval

**Figure 1:**
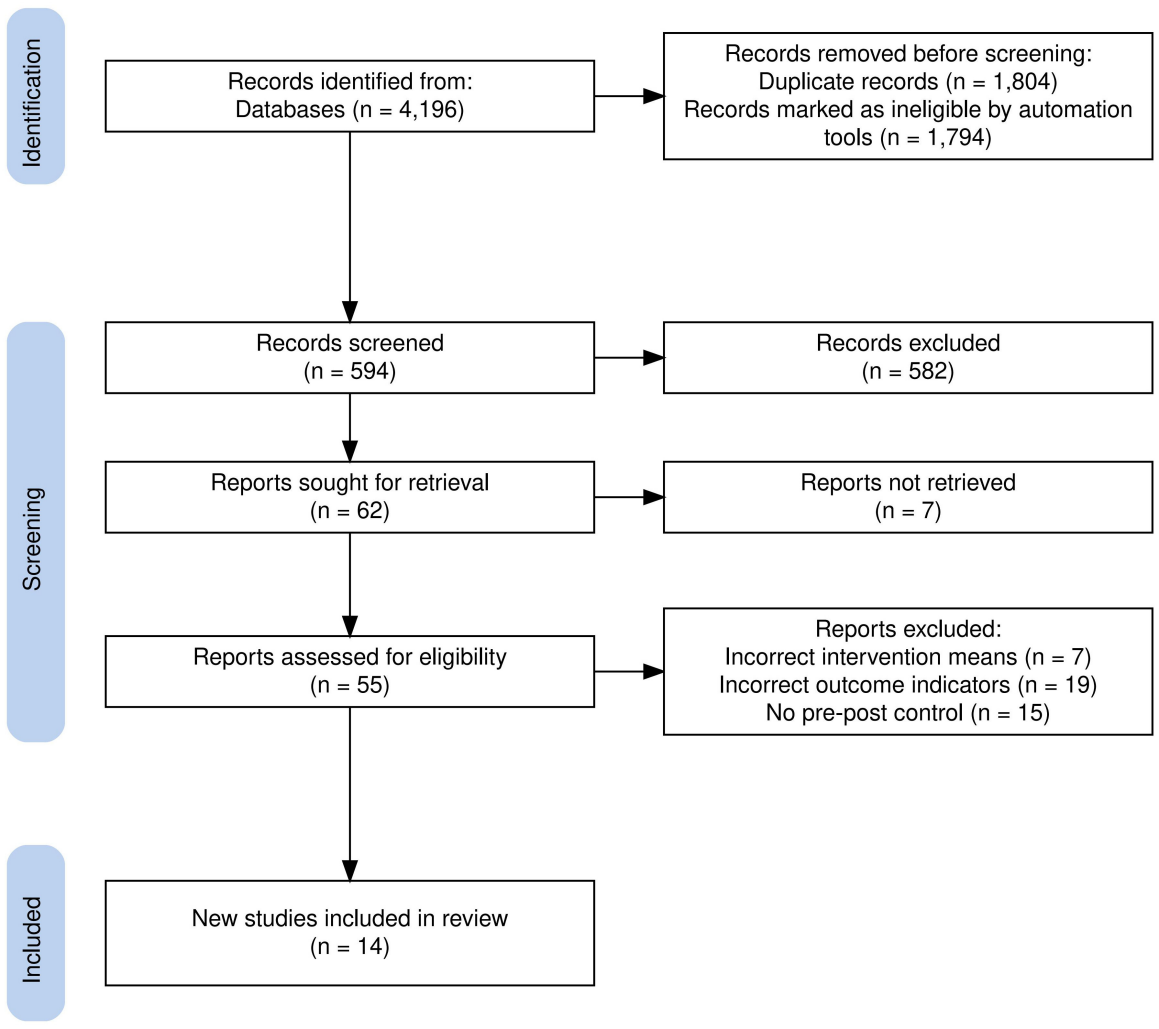
RCT Selection by PRISMA guidelines flow.

Two authors conducted a comprehensive search across PubMed, Embase, Web of Science, Google Scholar, Chinese National Knowledge Infrastructure (CNKI), China Biomedical Literature Database, and Wanfang Databases to identify RCTs examining the impact of music therapy on depression in college students. The systematic search encompassed literature published between January 1, 2005, and February 5, 2025. A combination of subject headings and free-text terms was utilized, with the search terms being: (“music” OR “music therapy” OR “sound therapy”) AND (“depression” OR “depressive symptoms” OR “mental health” OR “college students” OR “university students” OR “freshman”). Publications in English and Chinese were included. Furthermore, a review of the references in the identified RCTs and key reviews was performed to expand the search scope. Figure 1 shows the literature screening process.

### 2.2 Inclusion and exclusion criteria

Studies were selected based on the following PICOS framework (Amir-Behghadami and Janati, 2020): (1) Collegiate populations clinically validated depressive symptoms diagnoses. While our research focused on college students, the study by Li (2021) targeting teenager was included based on the following considerations: The study sample included 18 years old individuals (upper age limit of the 13–18 range), overlapping with the lower age range of the college student population; All intervention subjects were clinically diagnosed with depression, and the core symptom profiles are consistent with those observed in the college student population. The therapeutic mechanism of songwriting therapy (expressive creation + group support) demonstrates cross-age applicability. (2) Structured music therapy protocols (active engagement or receptive listening) administered ≥4 weeks, adjunctive to routine psychological counseling or pharmacological treatments. (3) Control groups receiving standard care without music-based interventions. (4) Quantifiable depression metrics using validated scales (SDS, BDI, or SCL-90 depression subscale) with pre-post score comparisons. (5) Peer-reviewed RCTs published in Chinese or English. Studies were excluded based on (1) Lack of standardized diagnostic procedures or concurrent comorbidities (other psychiatric disorders, neurological/endocrinal diseases). (2) High attrition rates (>30%) and inadequate randomization. (3) case series (<10 participants) or studies reporting composite scores without depression-specific data.

### 2.3 Literature selection and data retrieval

Two authors independently carried out the literature screening and data extraction in a blinded fashion, with discrepancies addressed through collaborative review and dialogue. The information collated from the studies encompassed the authorship, year of publication, country, Object characteristics, Sample size, Therapy approach, Intervention cycle, and the outcomes reported. The core metrics of interest were the Self-rating Depression Scale (SDS), Symptom Checklist 90 (SCL-90), and Beck Depression Inventory (BDI). These were selected to thoroughly evaluate how music therapy affects depressive symptoms in college students, forming a solid basis for the meta-analysis.

### 2.4 Features of the included studies

A total of 16 RCTs were included, involving 938 participants. Among them, 472 patients received music therapy, while 461 patients received conventional drug treatment or did not receive music—therapy interventions. The basic information of the included literatures is shown in Table 1.

**Table 1:** Basic information of included RCTs on music therapy.

**Study ID**	**Country**	**Object characteristics**	**Sample size**		**Therapy approach**		**Intervention cycle**	**Scale**
			**Music group**	**Control group**	**Music group**	**Control group**		
Ghasemi et al. (2017)	Iran	Students in the faculty of dentistry	44	44	Music therapy (with at least 1-year of music practice history)	No music practice experience	Not less than 4 weeks	BDI
Putri (2024)	Indonesia	Young adult depression patients	50	50	Music therapy	Conventional drug treatment	Once every 4 weeks, follow-up time points are 3 months and 6 months	BDI
Zhang M. et al. (2022)	China	Undergraduates aged 18–20	36	35	Group Improvisational Music Therapy (GIMT)	No GIMT intervention	A total of 4 weeks, once a week	BDI
Wan (2013)	China	Undergraduates	24	24	Orff group music therapy	No intervention	A total of 10 times, twice a week, 1.5 h each time	SCL-90
Wang et al. (2020)	China	College students majoring in clinical medicine with depression	33	33	Music therapy	No intervention	A total of 2 months, once a week	SDS SCL-90
Zhang et al. (2014)	China	College students with depressive states	50	32	Chinese Five - element Music Therapy	No music therapy	A total of 6 weeks, twice a week, 30 min each time	SDS SCL-90
Chen (2005)	China	Undergraduate students in medical colleges	33	37	Music relaxation training	No intervention, only measurement and evaluation at the same time as each intervention group	A total of 4 weeks	SCL-90
Wang and Ding (2013)	China	College students with depressive tendencies	15	15	Music therapy + social dance	Practice other elective courses	A total of 11 weeks	SDS
Shan et al. (2012)	China	Freshmen in a traditional Chinese medicine hospital	30	30	Chinese Five - element Music Therapy	No intervention, mainly follow-up	A total of 30 weeks	SCL-90
Wang (2007)	China	College students with social anxiety	48	48	Group music therapy	No music therapy, in normal study state	A total of 8 weeks, once a week, 60 min each time	SCL-90
Chen et al. (2010)	China	Freshmen in a medical college	30	30	Music therapy + group counseling	Group counseling	8-week treatment course	SDS
Yu (2009)	China	Undergraduate students in the English Department of a foreign language college	35	37	Music aesthetic appreciation activities	No music aesthetic appreciation activities	A total of 3 months	SCL-90
Zhou (2005)	China	Undergraduates	31	31	Music aesthetic appreciation activities	No music aesthetic appreciation intervention	A total of 3 months, once a week, 9 times in total	SDS
Li (2021)	China	Adolescent patients with depressive disorders	13	15	Therapeutic songwriting + conventional drug treatment	Conventional drug treatment	A total of 1 month, twice a week, 1.5 h each time	SDS

### 2.5 Methodological quality appraisal

**Figure 2:**
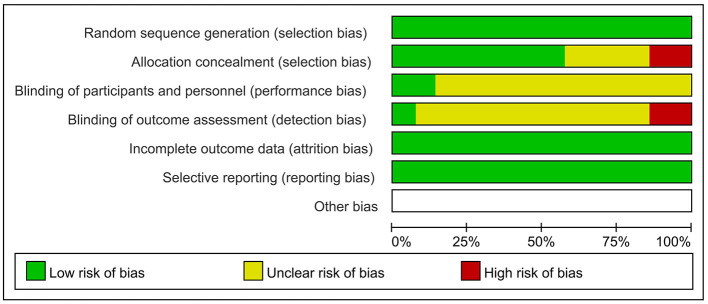
Risk of bias graph.

**Figure 3:**
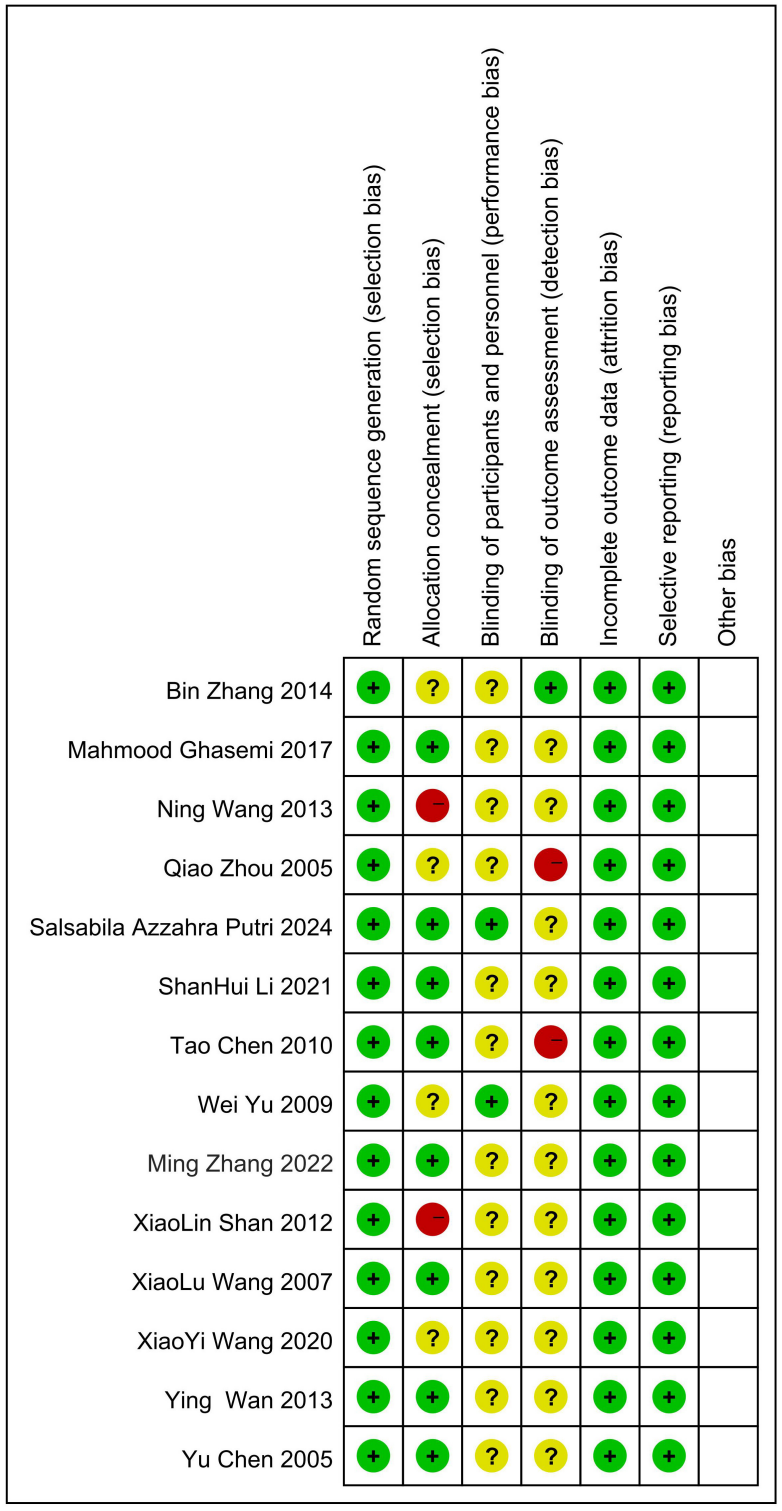
Risk of bias summary.

Two authors used the risk-of-bias tool recommended by the Cochrane Library (Higgins et al., 2011) to assess the quality of the included studies. This tool evaluates studies across several aspects: random sequence generation, Allocation concealment, Blinding of participants and personnel, Blinding of outcome assessment, Incomplete outcome data, Selective reporting of outcomes, and any “other” relevant issues. For each domain, studies are categorized as having “low risk of bias,” “unclear risk of bias,” or “high risk of bias.” The quality of the included RCTs was evaluated through Figures 2, 3, and the overall quality was good. High risk of bias was observed in allocation concealment and blinding (Figure 2). This limits generalizability but reflects music therapy trial challenges.

### 2.6 Quantitative synthesis protocol

Review Manager (RevMan) Version 5.3 software was employed for the meta-analysis. Data were presented as mean differences with their corresponding 95% confidence intervals (CI). To assess heterogeneity, we utilized the *Q* test alongside the *I*^2^ statistic for a quantitative evaluation. In cases where studies showed homogeneity (*I*^2^ <50%), a fixed—effects model was applied to compute the pooled estimates. Conversely, when heterogeneity was present (*I*^2^ ≥ 50%), a random-effects model was deemed appropriate. Sensitivity analyses were conducted by sequentially omitting individual studies and recalculating the pooled results to assess the robustness of the findings. To evaluate publication bias, forest plots were constructed and Egger's test was applied. In the analysis, the threshold for statistical significance between groups was a *p*-value of <0.05.

## 3 Meta-analysis results

### 3.1 Effect of music therapy on SDS depression scores

**Figure 4:**
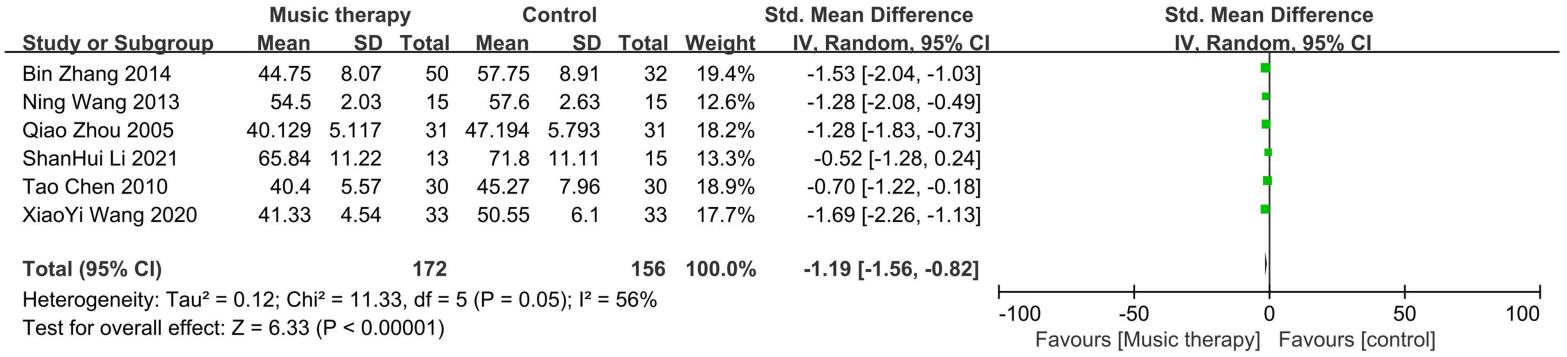
Forest plot for SDS scores.

**Figure 5:**
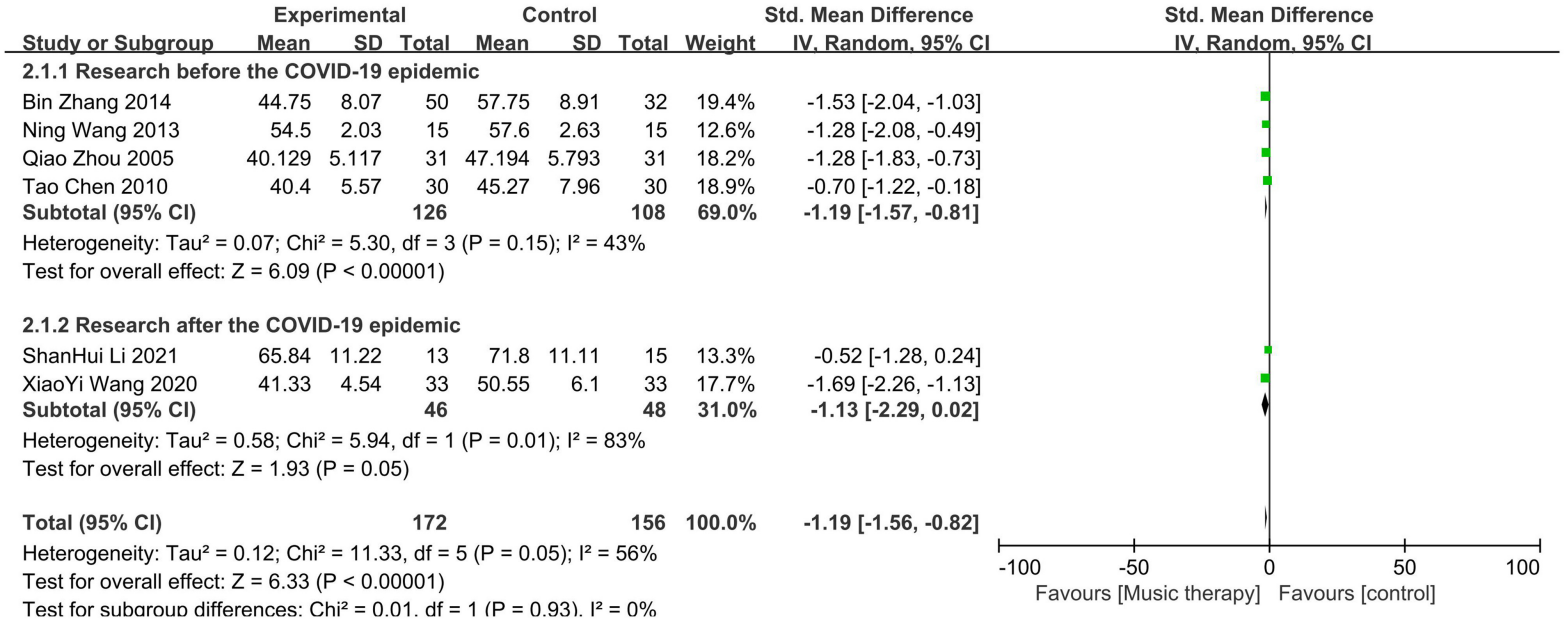
Subgroup analyses for SDS scores.

In six studies there were significant differences in the mean depression scores between the music therapy group and the control group, with the music therapy group showing a reduction in scores. It is worth noting that five of these studies focused on college students, who are known to experience higher levels of depression due to academic pressure and life transitions. Given that depression can have its roots in earlier stages of education, one study also targeted high school students as a preventive measure to address potential mental health issues before they escalate in college. This high school study was conducted by Li (2021). A random-effects model was used to meta-analyze the differences in SDS scores between the experimental and control groups, as shown in Figure 4. The overall effect size for the six studies was −1.19, with a 95% confidence interval of (−1.56, −0.82), indicating that music therapy is effective in reducing depression. Significant heterogeneity was among the studies (*I*^2^ = 56%, *P* = 0.05). Subgroup analyses are presented in Figure 5.

### 3.2 Effect of music therapy on SCL-90 depression scores

**Figure 6:**
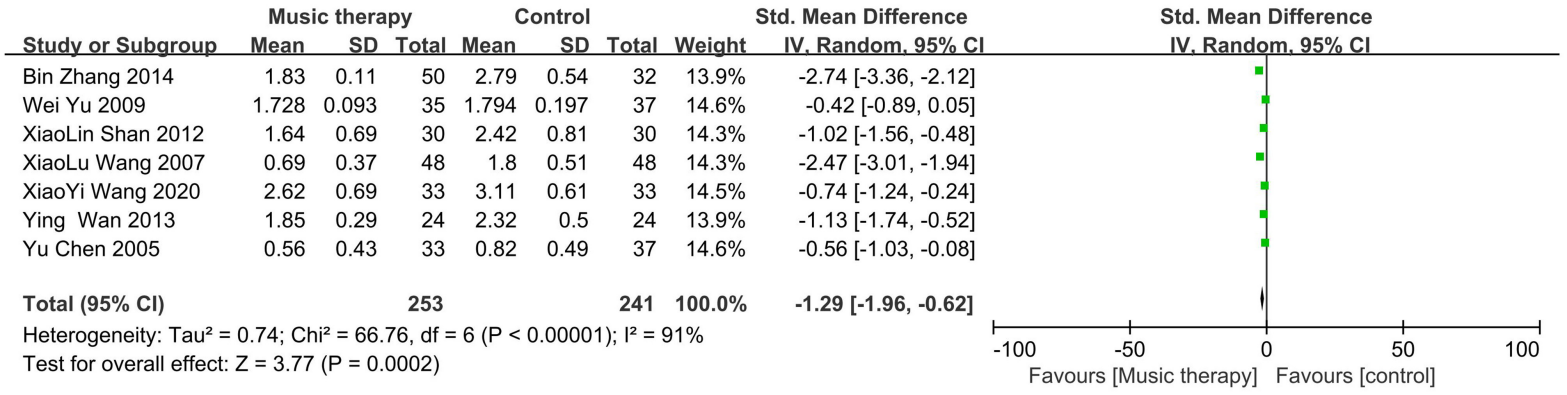
Forest plot for SCL-90 scores.

**Figure 7:**
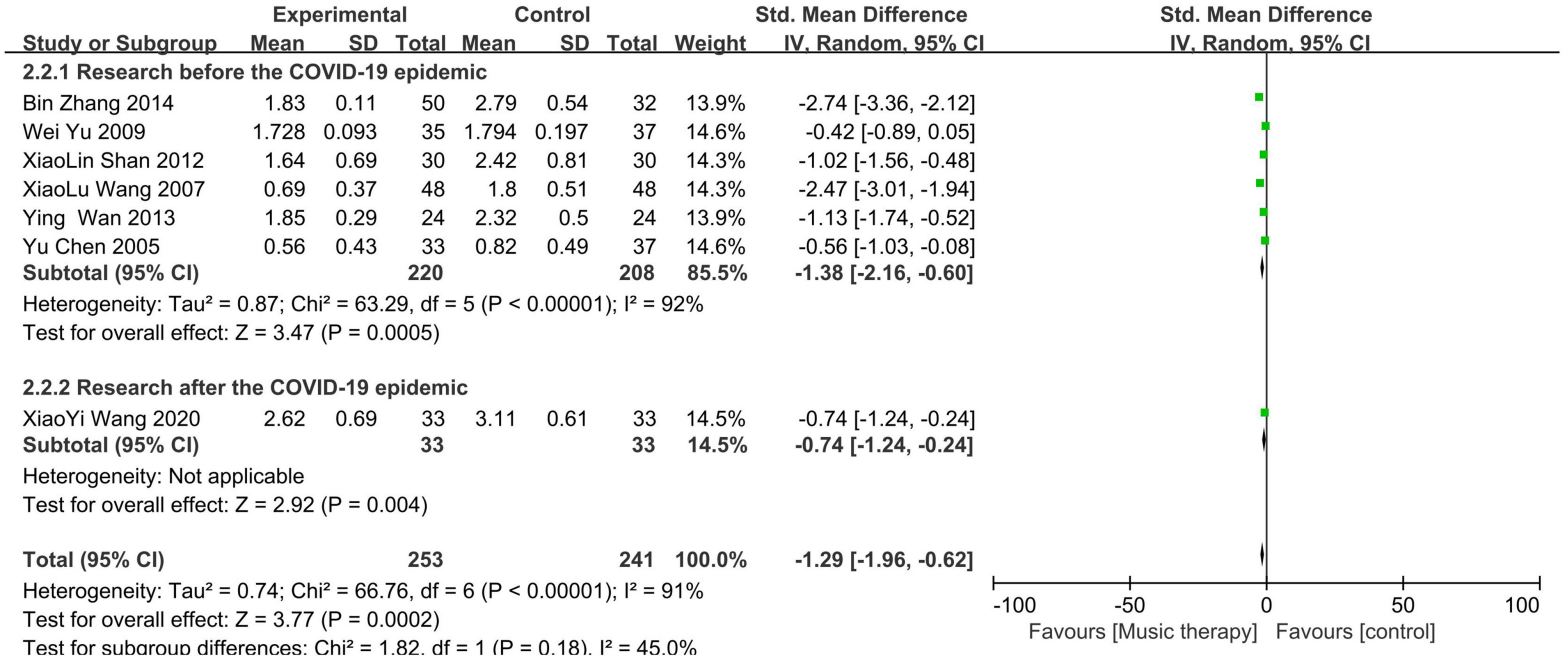
Subgroup analyses for SCL-90 scores.

Apart from one study (Yu, 2009), the mean depression scores were significantly different between the music therapy group and the control group in the other six studies, with the music therapy group having lower scores. A random-effects model was used to meta-analyze the differences in SCL-90 scores between the experimental and control groups, as shown in Figure 6. The overall effect size for the seven studies was −1.29, with a 95% confidence interval of (−1.96, −0.62), indicating that music therapy is effective in reducing depression. However, the studies had substantial heterogeneity (*I*^2^ = 91%, *P* < 0.00001). Subgroup analyses are presented in Figure 7.

### 3.3 Effect of music therapy on BDI depression scores

**Figure 8:**
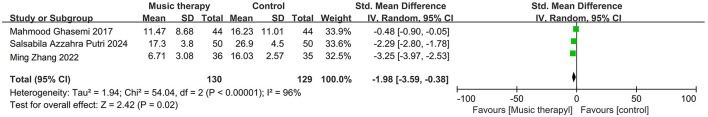
Forest plot for BDI scores.

**Figure 9:**
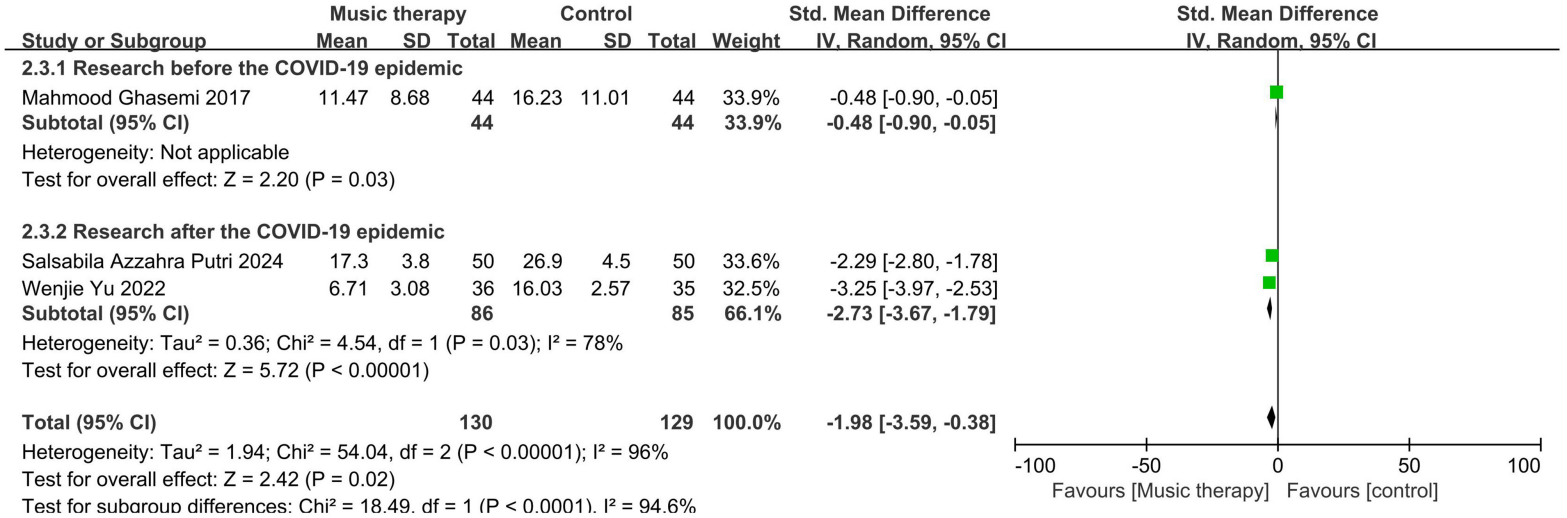
Subgroup analyses for BDI scores.

In three studies, there were significant differences in the mean depression scores between the music therapy group and the control group, with the music therapy group showing a reduction in scores. A random-effects model was used to meta-analyze the differences in BDI scores between the experimental and control groups, as shown in Figure 8. The overall effect size for the three studies was −1.98, with a 95% confidence interval of (−3.59, −0.38), indicating that music therapy is effective in reducing depression. There was significant heterogeneity among the studies (*I*^2^ = 96%, *P* < 0.00001). Subgroup analyses are presented in Figure 9.

## 4 Discussions

This meta-analysis included 16 studies. The pooled effect size for the six studies using the SDS was −1.19 (95% CI: −1.56, −0.82), indicating lower scores in the treatment group compared to the control group, with moderate heterogeneity that improved in subgroup analyses. For the seven studies using the SCL-90, the pooled effect size was −1.29 (95% CI: −1.96, −0.62), again favoring the treatment group but with substantial heterogeneity that persisted after subgroup analyses. Similarly, the three studies using the BDI showed a pooled effect size of −1.98 (95% CI: −3.59, −0.38), with significant heterogeneity unmitigated by subgroup analyses.

The pooled effect sizes across measurement tools consistently favored music therapy, yet the substantial heterogeneity observed warrants cautious interpretation. For SCL-90 (*I*^2^ > 75%) and BDI (*I*^2^ > 80%), heterogeneity persisted despite subgroup analyses, suggesting methodological and clinical diversities beyond conventional moderators. Key limitations include: (1) Variability in intervention protocols potentially diluting dose-response consistency; (2) Control group disparities inflating effect sizes in uncontrolled designs.

Among the 16 studies, three demonstrated particularly notable effect sizes: those by Wang Xiaolu, Zhang Bin, and Zhang Ming. Wang's (2007) reported a significant effect size (*d* = −2.47), potentially attributable to the inclusion of the “*College Students' Music Preference Questionnaire.”* This tool comprehensively assessed participants' musical literacy and preferences, enabling personalized music selection aligned with individual characteristics. Such tailored interventions likely enhanced engagement and therapeutic relevance, thereby amplifying the effect size. This innovative approach underscores the importance of integrating participant preferences into music therapy design.

Zhang et al.'s (2014) achieved an effect size (*d* = −2.74), potentially due to the application of Traditional Chinese Medicine Five Elements Music Therapy. Music was selected according to each participant's condition and personal preference to address their depression. The music therapy was carried out in a dedicated room. The room was ensured to be quiet with a well-controlled temperature to enable the participants to engage fully. Discussions with the participants before and after the therapy refined the treatment protocols. These refinements contributed to the observed effectiveness of the therapy.

Zhang et al.'s (2022) reported the largest effect size (*d* = −3.25), likely stemming from rigorously designed RCTs of Group Improvisational Music Therapy (GIMT). GIMT specifically addressed emotional dysregulation and depressive symptoms. The Random allocation made sure that the groups were comparable at the start. The professional therapeutic setting created a great environment for participants to fully engage in musical experiences. Semi-structured interviews were carried out, offering qualitative insights. These insights worked together with the quantitative results, improving the accuracy of how the effects were evaluated.

The included studies used a wide range of music therapy methods for this meta-analysis. Each method had its own way of using music to address the research goals, which added richness to the overall analysis of how music therapy impacts the relevant outcomes. These methods encompassed different aspects of music therapy, such as receptive, recreative, and improvisational techniques, along with the unique Traditional Chinese Medicine Five Elements Music Therapy. Receptive methods, like music relaxation and imagery, focused on the patients' passive experience of music. Music relaxation calms patients by allowing them to listen to gentle and calming music, helping them decompress and reduce stress, on the other hand, Music imagery encouraged patients to create mental images while listening, facilitating an exploration of their inner emotions including singing and instrument playing, enabled patients to actively engage with music. Singing provided an outlet for emotional expression, while instrument playing enhanced creativity and self-confidence. Improvisational techniques, such as rhythm exercises, Guided Imagery, and Music Therapy, promoted spontaneous musical creation and self-discovery. Additionally, Traditional Chinese Medicine Five Elements Music Therapy was incorporated, which is based on the ancient Chinese philosophy of the five elements and their relationships with different musical tones. This therapy aimed to balance the body's energy and emotions through specific musical compositions.

The diversity in methodological approaches stemmed from two key factors. On one hand, it showed how adaptable the interventionists were. These were mostly graduate students in psychology and education. Their study in these areas gave them the know-how to understand patients' psychological needs. Being young and full of energy, they were also willing to try new and creative therapy methods. They were keen to test different music therapy techniques to get the best outcomes. On the other hand, it was a way to match the cognitive skills and musical interests of college students. College students have fairly advanced thinking skills. This let them get really involved in different music therapy methods. Since they were very interested in music, they were more likely to take an active part in therapy sessions. This reciprocal engagement enhanced treatment efficacy and shaped interventionists' methodological choices, ultimately yielding a wide array of music therapy approaches in the included studies.

Beyond methodological diversity, music therapy exerts its effects via multimodal therapeutic mechanisms: (1) Neuroaffective Regulation: improvisational techniques (Zhang X. et al., 2022) stimulate prefrontal-amygdala circuitry, enhancing emotional processing akin to mindfulness-based interventions (Kölsch, 2021). (2) Psychosocial Engagement: recreative methods (e.g., instrument playing) foster self-efficacy through mastery experiences, paralleling CBT's behavioral activation component (Harpaz and Vaizman, 2023).

When juxtaposed with established non-pharmacological interventions, music therapy demonstrates distinct advantages: (1) Versus CBT: While CBT shows larger pooled effects for depression (*g* = −0.79; Cuijpers et al., 2023), music therapy offers superior acceptability (dropout rates: 8% vs. 15% in CBT trials). (2) Versus Exercise Therapy: Music's immediate anxiolytic effects (−1.29 vs. exercise's −0.48 at 4 weeks; Schuch et al., 2021) better address acute distress, though exercise sustains longer-term neurotrophic benefits.

In music therapy practice, the therapeutic relationship is a crucial determinant of outcomes. Four studies (Zhou, 2005; Li, 2021; Yu, 2009; Wang, 2007) explicitly integrated participants' musical preferences, leveraging familiar music to enhance emotional induction and strengthen the rapport between therapists and participants. Additionally, four studies (Putri, 2024; Wang et al., 2020; Chen et al., 2010; Chen, 2005) centered on medical students, a subgroup confronted with intensified stressors such as academic pressure, limited social support, and challenges in clinical training. These factors contribute to higher depression rates among medical students compared to the general student population, particularly during clinical rotations. Consequently, integrating music therapy into medical education could serve as a vital component of mental health support, alleviating depressive symptoms and enhancing wellbeing (Aalbers et al., 2022).

The innovative methodologies in high-effect studies (e.g., Wang's personalized music selection; Zhang B's TCM integration) underscore the “active ingredients” of music therapy: participant-specific adaptation and multisensory engagement. Medical students' pronounced response (Putri, 2024; Chen et al., 2010) highlights its utility for high-stress subgroups where traditional counseling faces barriers.

Persistent heterogeneity suggests future trials should: (1) Standardize “dose” metrics (e.g., ISO-defined sound energy density); (2) Incorporate biomarker assessments (e.g., HRV, IL-6) to quantify physiological mechanisms; (3) Conduct network meta-analyses comparing music therapy variants against other interventions.

In summary, the breadth of music therapy approaches in this meta-analysis highlights both the methodological creativity of researchers and the alignment of interventions with participants' unique needs. Such versatility provides a robust foundation for exploring music therapy's role in emotional regulation and depression mitigation, particularly among college students (Zhang M. et al., 2022).

## 5 Methodological considerations and limitations

Most studies emphasized rigorous environmental controls (Zhang et al., 2014; Zhou, 2005; Wang, 2007; Chen, 2005) including quiet settings and optimized sound levels, to minimize external noise—a practice essential for ensuring internal validity. Future research should continue refining such protocols to enhance reproducibility.

However, several limitations warrant attention. Few studies reported robust randomization procedures, allocation concealment, or blinding (though double-blinding remains challenging in music therapy). Attrition rates and reasons for dropout were often omitted, potentially biasing results (Lee, 2019). To enhance credibility, future trials should adopt CONSORT-compliant reporting, implement evaluator blinding, and transparently address attrition (Turner et al., 2012).

## 6 Conclusion

Music therapy shows promising efficacy in alleviating depressive symptoms, mitigating academic anxiety, enhancing sleep quality, and promoting emotional expression among college students. Its accessibility, cost-effectiveness, and high acceptability position it as a valuable, practical component within university mental health support programs, offering a scalable and non-invasive intervention to address the growing psychological needs of the student population. Future research should expand outcome assessments to include diverse psychological variables (e.g., cognitive function, resilience) and explore mechanisms underlying therapeutic effects. Methodological rigor, including stratified randomization and mixed-methods designs, will further solidify the evidence base. By addressing these gaps, music therapy can be optimized as a scalable, non-invasive intervention within China university mental health programs.

Future clinical trials must prioritize methodological rigor to solidify the evidence base. This includes employing stratified randomization, robust blinding procedures (where feasible), longitudinal assessments to evaluate sustained effects, standardization of intervention protocols, and mixed-methods designs to capture both quantitative outcomes and qualitative experiences. By addressing these gaps and enhancing methodological quality, music therapy can be effectively optimized and integrated into comprehensive mental health strategies within university settings.

## Data Availability

The aggregated data supporting this meta-analysis are included in the article, further inquiries can be directed to the corresponding author.
